# Oral rehydration solution coverage in under 5 children with diarrhea: a tri-country, subnational, cross-sectional comparative analysis of two demographic health surveys cycles

**DOI:** 10.1186/s12889-020-09811-1

**Published:** 2020-11-16

**Authors:** Philimon N. Gona, Clara M. Gona, Vasco Chikwasha, Clara Haruzivishe, Sowmya R. Rao, Chabila C. Mapoma

**Affiliations:** 1 College of Nursing & Health Sciences, University of Massachusetts Boston, 100 Morrissey Boulevard, Boston, MA 02125 USA; 2 Department of Nursing, MGH Institute for Health Professions, Boston, MA USA; 3 University of Zimbabwe College of Health Sciences, Harare, Zimbabwe; 4 Department of Global Health, Boston University Medical School, Boston, MA USA; 5 Department of Population Studies, University of Zambia, Lusaka, Zambia

**Keywords:** Oral rehydration solution, Under 5 children, Diarrhea, Demographic health surveys

## Abstract

**Background:**

More than 3 million children under 5 years in developing countries die from dehydration due to diarrhea, a preventable and treatable disease. We conducted a comparative analysis of two Demographic Health Survey (DHS) cycles to examine changes in ORS coverage in Zimbabwe, Zambia and Malawi. These surveys are cross-sectional conducted on a representative sample of the non-institutionalized individuals.

**Methods:**

The sample is drawn using a stratified two-stage cluster sampling design with census enumeration areas, typically, selected first as primary sampling units (PSUs) and then a fixed number of households from each PSU. We examined national and sub-regional prevalence of ORS use during a recent episode of diarrhea (within 2 weeks of survey) using DHSs for 2007–2010 (1st Period), and 2013–2016 (2nd Period). Weighted proportions of ORS were obtained and multivariable- design-adjusted logistic regression analysis was used to obtain Odds Ratios (aORs) and 95% confidence intervals (CIs) and weighted proportions of ORS coverage.

**Results:**

Crude ORS coverage increased from 21.0% (95% CI: 17.4–24.9) in 1st Period to 40.5% (36.5–44.6) in 2nd Period in Zimbabwe; increased from 60.8% (56.1–65.3) to 64.7% (61.8–67.5) in Zambia; and decreased from 72.3% (68.4–75.9) to 64.6% (60.9–68.1) in Malawi. The rates of change in coverage among provinces in Zimbabwe ranged from 10.3% over the three cycles (approximately 10 years) in Midlands to 44.2% in Matabeleland South; in Zambia from − 9.5% in Eastern Province to 24.4% in Luapula; and in Malawi from − 16.5% in the Northern Province to − 3.2% in Southern Province. The aORs for ORS use was 3.95(2.66–5.86) for Zimbabwe, 2.83 (2.35–3.40) for Zambia, and, 0.71(0.59–0.87) for Malawi.

**Conclusion:**

ORS coverage increased in Zimbabwe, stagnated in Zambia, but declined in Malawi. Monitoring national and province-level trends of ORS use illuminates geographic inequalities and helps identify priority areas for targeting resource allocation.. Provision of safe drinking-water, adequate sanitation and hygiene will help reduce the causes and the incidence of diarrhea. Health policies to strengthen access to appropriate treatments such as vaccines for rotavirus and cholera and promoting use of ORS to reduce the burden of diarrhea should be developed and implemented.

**Supplementary Information:**

The online version contains supplementary material available at 10.1186/s12889-020-09811-1.

## Background

Diarrhea is the second leading cause of death and malnutrition in children under 5 years (U5) of age annually responsible for more than 500,000 deaths globally [1]. Most deaths due to diarrhea are dehydration related. Dehydration causes the body to lose water and salts necessary for survival [2]. .Goal 3 of the UN 2030 Agenda for Sustainable Development Goals (SDGs), seeks “to ensure healthy lives and promote wellbeing for all at all ages”. SDG Target 3.2 aims to end preventable deaths of newborns and children under 5 years, by 2030, to reduce neonatal mortality to less than 12 deaths per 1000 livebirths, and under-5 mortality to be lower than 25 deaths per 1000 live births [1, 3].

Repeated bouts of diarrhea weaken children contributing to protein-energy malnutrition. Oral rehydration solution (ORS), also known as oral rehydration therapy (ORT) has significant potential to drastically reduce child deaths caused by dehydration and under-nutrition in children with diarrhea. ORS for diarrhea prepared and used at home has been dubbed “the most important medical advance of this century” [4] annually saving over 1 million children under 5 years over the past 25 years globally [3]. The World Health Organization (WHO) [5], and the United Nations Children’s Fund (UNICEF) [6] have promoted use of ORS as an essential medicine to treat diarrhea [7]. UNICEF estimates from 2000 suggest that only 34% of children under 5 years in low- and middle-income countries (LMICs) received ORS to treat diarrhea; however, coverage increased to 44% in 2016, implying that majority of children under 5 with diarrhea were not treated [5, 6]. .ORS is especially suitable in locations where intravenous fluids are readily available [8].

Mothers are the first line of defense in administering ORS where contents of packets containing standardized premade sodium and glucose are dissolved in one liter of clean water [9]. .However, UNICEF estimated that fewer than half the children under 5 with diarrhea in LMICs received ORS in 2017. A 2010 meta-analysis estimated that 100% coverage of ORS could prevent 93% of diarrheal deaths [10]. .Coverage of ORS remains low despite inclusion in the WHO Essential Medicines List (EML) and Global Action Plan for the Prevention and Control of Pneumonia and Diarrhea (GAPPD) [11–13].

**Fig. 1:**
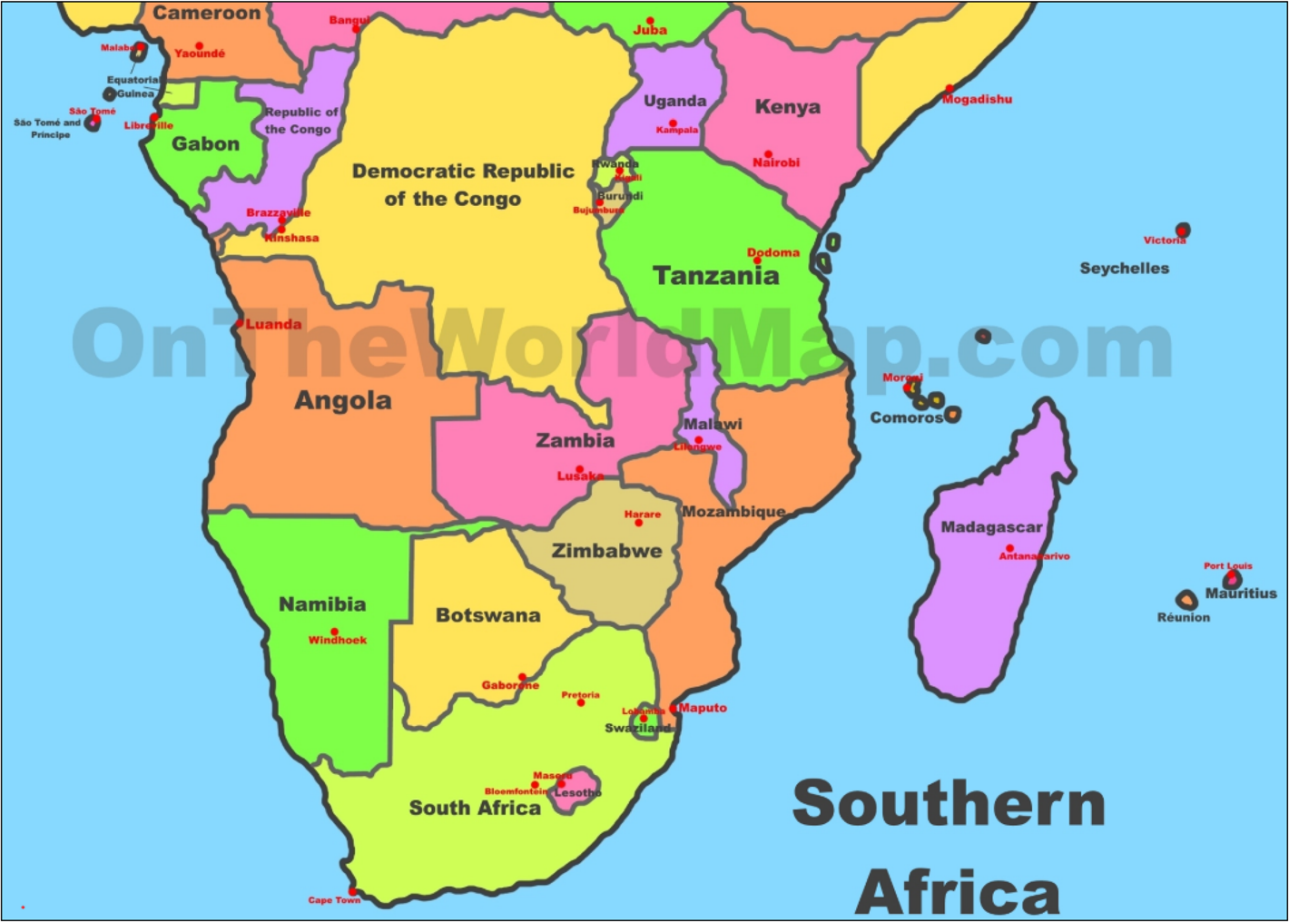
Map of Zimbabwe Zambia and Malawi, formerly known as Central African Federation from 1953 to 1963. Copyright: OneWorldMap http://ontheworldmap.com/africa/map-of-southern-africa.jpg. Besides sharing a common British colonial history with the three countries belonging to the Federation of Rhodesia, and Nyasaland. Known as the Central African Federation, the Federation of Rhodesia and Nyasaland was created in1953, and lasted until 1963. The federation joined the British protectorate of Northern Rhodesia (now Zambia), the colony of Southern Rhodesia (now Zimbabwe), and the protectorate of Nyasaland (now Malawi)

**Fig. 2:**
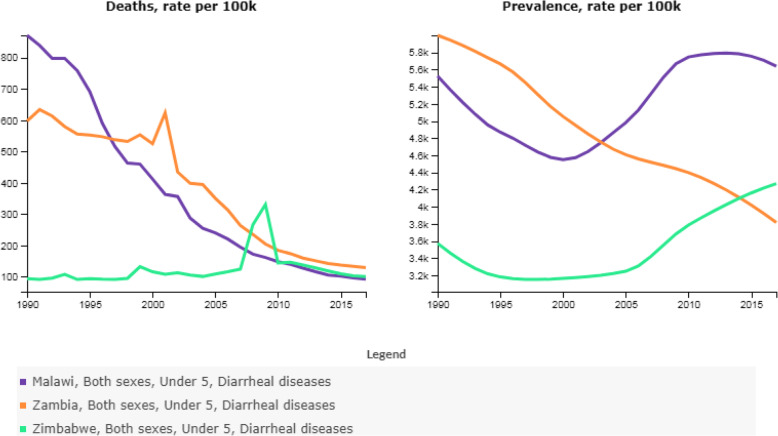
Death rates and prevalence per 100,000 population of diarrheal disease in children under 5 years, from 1990 to 2017 in Zimbabwe, Zambia and Malawi. [Purple] Malawi, Both sexes, Under 5, Diarrheal diseases. [Orange] Zambia, Both sexes, Under 5, Diarrheal diseases. [Green0 Zimbabwe, Both sexes, Under 5, Diarrheal diseases. Source: http://ghdx.healthdata.org/gbd-results-tool?params=gbd-api-2017-permalink/d84a532278e2428bdd5e1106ee976634

The efficacy of ORS in U5 will boost confidence in attaining SDG Target 3.2 but to our knowledge, for Zimbabwe, Zambia, and Malawi (Fig. 1), there have been no studies to estimate subnational coverage of ORS and the drivers for low coverage. We sought to estimate ORS coverage over space and time in three neighboring LMICs, Zimbabwe, Zambia and Malawi, and examine geographic inequalities within the three countries. We used publicly available data collected by the Demographic Health Surveys (DHS) [14]. The burden of diarrhea in the three countries from 1990 to 2017 is shown in Fig. 2 [15]. In 2017, diarrheal disease caused 129.3(95% uncertainty interval [UI]:77.5–184.0) in Zambia, 100.6(66.2–141.3) in Zimbabwe, and 92.6 (60.7–134.6) per 100,000 in Malawi compared to 78.4 (70.1–87.1) globally. The corresponding rate of years of lives lost (YLLs) per 100,000 population in the same year was 11,204 (7592-15,924) for Zambia, 8722 (5729-12,262) for Zimbabwe, and 8024(5257-11,663) for Malawi, representing high endemic areas [15].

While county-to-county variation exists in both the likelihood of a child experiencing a diarrhea episode resulting in death, greater variations exist at provincial or subnational level. To reduce the public health burden of childhood diarrhea and identify risk factors that require targeted interventions to alleviate disease burden, countries and subnational regions with the highest prevalence and those with the lowest levels of ORS coverage should be identified. Similarly, studies have shown considerable variation between countries in ORS use; however subnational variations are currently unknown [7]. Quantification of both the local coverage of ORS and its drivers is critical to inform resource allocation and the effectiveness of interventions. Country-level evaluation of these determinants of ORS coverage can mask subnational variation and dilute the critical information needed to inform or formulate policy. A sub-national region or location with a small population within a country, for example, may have a relatively high ORS coverage, but a sufficiently large sub-national region may be a greater contributor to low ORS coverage overall, thus, decisions aimed at improving ORS coverage may overlook those at highest risk [16]. The need therefore to design intervention strategies that efficiently save lives while also highlighting entrenched geographic disparities is critical. Thus, our goal was to examine changes in patterns of province-level ORS coverage over two DHS Cycles (2007/2010 and 2013/2016) in three countries of Zimbabwe, Zambia and Malawi to help identify subnational areas in need of targeted interventions.

## Methods

A stratified two-stage cluster sampling design was used first to select primary sampling units (PSUs) and secondly to select a fixed number of households from each PSU. The surveys are conducted by interviewers approximately every 5 years to provide data for monitoring and evaluation of indicators for population health and to provide current demographic and health information for use by policymakers, planners, researchers and program managers. The demographic health surveys collect systematic and have comparable data across countries. These surveys are designed to yield representative information for most of the indicators for the country and are designed to cover 100% of the target population in the country. So that exclusions are not encountered during field work, households or dwellings to be excluded are pre-specified and pre-identified to not be included in the final list of the households in the selected EAs. Institutional living arrangements such as army barracks, hospitals, police camps, and boarding schools are excluded from the frame. All these decisions were made at the very beginning of the survey, before the sample is drawn. The survey interviewers then interviewed only the preselected households. No replacements and no changes of the pre-selected households are allowed in the implementation stages [14].

### Sampling design

The sample is drawn using a stratified two-stage cluster sampling design. The primary sampling unit (PSU), typically census enumeration areas (EA), are selected with probability proportional to size within each stratum. A fixed number of households is then selected by equal probability systematic sampling in the selected EAs. An eligible woman aged 15–49 in each household is then selected to respond to the Women’s survey [14].

### Questionnaires

DHS surveys collect data through four main interviewer-administered questionnaires. The Household Questionnaire collects information on the characteristics of the household and a list all household members. The household roster within this questionnaire captures key characteristics of each household member and is used to select women and men eligible for individual interviews. The Woman’s Questionnaire, in addition to questions about the woman, contains a birth history which is then used to list all children (alive or dead) that the respondent has given birth to and the child’s survival status as well as caregiver knowledge of diarrhea care and treatment for diarrhea.. The questionnaires and the survey procedures followed in each country are similar resulting in comparable information, dataset filenames, variable types, names, and coding across countries [14].

### Survey protocols

The months prior to the survey are devoted to planning, survey logistics, sample design, questionnaire design, household listing, pretest, revision of questionnaires and manuals, training of field personnel, data processing set up, and fieldwork.. HIV testing protocol provides for informed, anonymous, and voluntary testing. Since the testing is anonymous, survey respondents cannot be provided with their results.

### Data processing and curation

The DHS Program uses a software package, CSPro (see www.census.gov/data/software/cspro.html), to process its surveys. CSPro is developed by the US Bureau of the Census, ICF, and SerPro SA. CSPro is used in The DHS Program in all steps of data processing with no need for another package or computer language. All steps, from entering/capturing the data to the production of statistics and tables published in DHS final reports, are performed with CSPro. The data is downloadable from https://dhsprogram.com/. We downloaded the data in SAS and Stata format for statistical analysis [14].

### Outcome measures

Our main outcome of interest was the proportion of U5 children with diarrhea who received ORS. The operational definition of ORS, therefore, was “a pre-packaged electrolyte solution containing glucose or another form of sugar or starch, as well as sodium, chloride, potassium, and bicarbonate” [10]. Diarrhea was defined as three or more abnormally loose or watery stools within a 24-h period. The following questions on the DHS Maternal questionnaire were used to determine whether ORS was administered or not during the most recent episode of diarrhea in children under 5:

*Now I would like to ask some questions about your children born in the last five years.*

*Has (NAME) had diarrhea in the last 2 weeks?*

*Was (NAME) given any of the following at any time since (NAME) started having the diarrhea?*

- *A fluid made from a special packet called an ORS sachet?*
- *A pre-packaged ORS liquid?*
- *A homemade sugar-salt-water solution (SSS)?*
- *Zinc tablets or syrup?*

Besides the answers to the above questions, no additional scoring was needed to determine whether a child was treated with ORS or not. The binary variable for ORS use in the last 2 weeks was used as the outcome variable in our statistical analysis.

### Independent variables

The DHS uses Principal Component Analysis to construct the household wealth index using a composite measure of a household’s cumulative living standard [17]. With inputs comprising of ownership of selected assets, such as televisions and bicycles; materials used for housing construction; and types of water access and sanitation facilities. The resulting asset scores are standardized with a mean of zero and a standard deviation of one. These standardized scores are then used to create the break points that define wealth quintiles as: Lowest, Second, Middle, Fourth, and Highest [17]. .Demographic data, i.e., the mother, age, education, HIV + status, geographic location (urban vs. rural) were obtained from the Woman’s Questionnaire.

### Statistical analysis

We obtained prevalence estimates along with 95% confidence intervals (CIs) at the two time periods-overall and subnational. We used the Chi-squared test to compare the prevalence at the two time periods. Furthermore, we computed prevalence estimates stratified by urban vs. rural, mother’s age (< 25 vs. ≥25 years), education (<high school vs. ≥ high school), HIV + status, and quintiles of household wealth index. For each Period, logistic regression analysis were conducted to obtain crude and multivariable-adjusted Odds Ratios (OR) and 95% CIs. The multivariable model adjusted for mother’s age, education, HIV status, urban vs. rural setting, subnational region and the period of survey. All statistical analyses were conducted using SAS/STAT v9.4 (SAS Institute Inc., Cary, North Carolina, USA) and Stata Software, Version 14.2 (StataCorp, College Station, Texas, USA) using sampling weights and accounted for the complex sampling design A two-sided *p*-value< 0.05 was considered significant.

DHS computes weight for a particular household as the inverse of its household selection probability multiplied by the inverse of the household response rate in the stratum and for an individual woman for a particular household as the inverse of its household selection probability multiplied by the inverse of the household response rate in the stratum. Optimal sample size for multi-stage stratified design like a two-stage cluster sampling design is dependent on intra-cluster correlation [18]. To estimate the composite outcome of “Child had diarrhea in the last 2-weeks” and “ Child treated with ORS” for 2015 Cycle for Zimbabwe, estimates from the previous cycles are assumed as the background estimate of prevalence and the sample design is also accounted for in the calculations. The background prevalence of ORS use for 2015 Cycle for Zimbabwe was 0.405, SE = 0.021, design effect = 1.303. A weighted sample of *n* = 1014 was sufficient to estimate with 95% confidence the prevalence of children treated with ORS to lie between 0.364–0.446, i.e., a margin of error (E) of 0.041. A similar approach was used for the DHS Cycles conducted in Zambia and Malawi [19].

Interviews are conducted only if the respondent provides voluntary informed consent. Procedures and questionnaires for standard DHS surveys have been reviewed and approved by ICF Institutional Review Board (IRB). Ministries of Health in individual countries provided ethical approvals and protection for human subjects: The Medical Research Council of Zimbabwe, the Tropical Disease Research Center Ethics Review Board of Zambia, and the National Health Sciences Research Committee of Malawi [14].

As part of a collaborative called the Federation of Rhodesia and Nyasaland, the countries share a past colonial history under Britain, and a present political and economic tie through a 16-member Southern Africa Development Community (SADC) (Fig. 1). SADC is a regional economic community whose aim is to increase regional socio-economic integration to achieve greater economic growth and poverty alleviation (www.sadc.int/about-sadc). The three countries have comparable socio-demographic index (SDI) measured by national wealth [18]. .The SDI, which ranges from 0 to 1 is a summary measure of where a location is on the spectrum of socio-demographic development. The index is calculated from the geometric mean of three rescaled components: total fertility rate (TFR) of women under 25 years of age, lag-distributed income per capita, and average educational attainment in the population > 15 years. SDI contains an interpretable scale: zero represents the lowest income per capita, lowest educational attainment, and highest TFR observed across all GBD geographies from 1980 to 2015, and one represents the highest income per capita, highest educational attainment, and lowest TFR [16]. The SDI for Zambia 0.47 (classified as low middle), for Zimbabwe 0.46, (low middle), and for Malawi 0.35, (low).

## Results

### National prevalence of ORS use

Tables 1A, 2A, and 3A show overall and subnational prevalence of ORS coverage during each period for the three countries. There were 688; 747; and 869 eligible mothers with children under 5 with diarrhea in 1st Period, and 1014; 1928; and 1153 in 2nd Period in imbabwe, Zambia, and Malawi, respectively. ORS coverage nearly doubled (19.5%) from 21.0 (17.4–24.9) to 40.5 (36.5–44.6), *p* < 0.001 in Zimbabwe, decreased in Malawi (7.7%) from 72.3 (68.4–75.9) to 64.6 (60.9–68.1), *p* = 0.004, but remained unchanged in Zambia from 60.8(56.1–65.3) to 64.7 (61.8–67.5), *p* = 0.156 (Tables 1, 3, and 4).

**Table 1:** Change in national and subnational prevalence of ORS use for Demographic Health Survey Cycles: Zimbabwe 2010/11 vs. 2015/16

Zimbabwe	2010/11,*N* = 688	2015/16, *N* = 1014	Change	*p*-value^a^
	P1% (CI)	P2% (CI)	P1 - P2	
**Overall**	21.0 (17.4–24.9)	40.5 (36.5–44.6)	19.5	< 0.001
**Province**				
Manicaland	19.5 (11.6–30.7)	36.7 (27.5–47.1)	17.2	0.019
Mashonaland Central	25.1 (15.7–37.7)	40.5 (28.6–53.7)	15.4	0.081
Mashonaland East	22.7 (14.0–34.6)	33.8 (24.1–45.1)	11.1	0.152
Mashonaland West	9.6(4.1–21.2)	36.1(26.4–47.1)	26.5	0.001
Matabeleland North	21.7 (13.5–32.9)	60.3 (45.8–73.2)	38.6	0.0001
Matabeleland South	12.3(5.4–25.7)	56.5(39.5–72.1)	44.2	0.0002
Midlands	28.4(19.0–40.3)	38.7(29.1–49.3)	10.3	0.186
Masvingo	14.1(7.2–25.8)	35.1(25.4–46.2)	21.0	0.008
Harare	27.7(16.5–42.7)	49.4(37.2–61.7)	21.7	0.028
Bulawayo	10.4(4.4–22.8)	50.3(33.7–66.8)	39.9	0.0002
**Location**				
Rural	18.4 (14.5–23.0)	37.8 (33.1–42.7)	19.4	< 0.001
Urban	25.9(19.2–34.1)	46.4 (39.1–53.9)	20.5	0.008
**Mother’s age**				
< 25 years	19.2 (14.4–25.1)	43.3 (36.7–50.1)	24.1	0.007
≥ 25 years	22.2(17.7–27.5)	38.9 (34.0–44.1)	16.7	0.010
**Education**				
< high school	14.9 (10.0–21.7)	32.6 (26.6–39.1)	17.7	0.011
≥ high school	24.2 (19.9–29.0)	44.7 (39.9–49.5)	20.5	0.006
**HIV status**				
HIV positive	22.8(14.8–33.40	41.0931.1–51.80	18.2	0.015
**Wealth Index (Quintile)**				
Lowest (1)	17.8(12.3–25.2)	29.7(22.8–37.7)	11.9	0.022
Second (2)	20.2(13.9–28.3)	37.0(29.2–45.5)	16.8	0.003
Middle (3)	17.5(10.5–27.6)	44.2(36.5–52.1)	26.7	0.0001
Fourth (4)	23.1(15.8–32.5)	49.5(42.1–57.0)	26.4	< 0.001
Highest (5)	27.6(18.6–38.7)	44.2(33.9–55.0)	16.6	0.031

^a^Based on a two-sided Chi-squared test

**Table 2:** National and subnational odds ratios for ORS use for Demographic Health Survey Cycles: Zimbabwe 2010/11 vs. 2015/16

Zimbabwe	2010/11, N = 688		2015/16, N = 1014	
	Crude OR(CI)	Adjusted OR(CI)	Crude OR(CI)	Adjusted OR(CI)
Overall	Ref	Ref	2.84(2.25–3.60)^a^	3.95(2.66–5.86) ^a^
**Region**				
Manicaland	0.44(0.15–1.33)	0.40(0.15–1.10)	0.97(0.52–1.82)	1.10(0.57–2.10)
Mashonaland Central	0.32(0.11–0.95)*	0.25(0.09–0.70)**	0.83(0.41–1.67)	0.87(0.43–1.76)
Mashonaland East	0.36(0.12–1.09)	0.29(0.11–0.78)*	1.11(0.57–2.14)	1.21(0.60–2.41)
Mashonaland West	Ref	Ref	Ref	Ref
Matabeleland North	0.39(0.13–1.14)	0.34(0.12–0.96)*	0.37(0.18–0.78)**	0.35(0.15–0.82)*
Matabeleland South	0.76(0.21–2.77)	0.71(0.20–2.55)	0.43(0.19–0.99)*	0.56(0.24–1.31)
Midlands	0.27(0.09–0.78)*	0.31(0.11–0.89)*	0.90(0.48–1.68)	1.11(0.57–2.16)
Masvingo	0.65(0.20–2.13)	0.51(0.17–1.50)	1.05(0.55–2.00)	1.10(0.54–2.23)
Harare	0.28(0.09–0.87)*	0.42(0.13–1.36)	0.58(0.29–1.14)	0.76(0.39–1.49)
Bulawayo	0.92(0.25–3.42)	0.94(0.20–4.40)	0.56(0.25–1.28)	0.76(0.31–1.86)
**Location**				
Rural	1.56(0.96–2.52)	1.48(0.65–3.34)	1.43(0.99–2.05)	0.78(0.43–1.39)
**Mother’s HIV status**				
HIV positive	0.84(0.47–1.51)	0.93(0.51–1.71)	1.05(0.66–1.68)	1.00(0.62–1.63)
**Wealth Index (Quintile)**				
Lowest (1)	1.76(0.90–3.43)	1.05(0.39–2.81)	1.87(1.06–3.30)*	1.72(0.82–3.63)
Second (2)	1.51(0.76–2.99)	0.85(0.31–2.35)	1.35(0.77–2.37)	1.42(0.68–2.97)
Middle (3)	1.80(0.84–3.87)	1.20(0.47–3.08)	1.00(0.58–1.72)	1.12(0.54–2.30)
Fourth (4)	1.27(0.63–2.54)	0.98(0.44–2.18)	0.81(0.49–1.33)	0.85(0.51–1.43)
Highest (5)	referent	referent	Referent	referent
**Mother’s age**				
< 25 years	1.21(0.78–1.86)	1.17(0.74–1.84)	0.84(0.59–1.18)	0.81(0.56–1.18)
≥ 25 years	Ref	Ref	Ref	Ref
**Education**				
< high school	1.82(1.09–3.03)*	1.51(0.86–2.66)	1.67(1.21–2.32)**	1.32(0.94–1.86)
≥ high school	Ref	Ref	Ref	Ref

^a^Corresponding to adjusted ORS coverage proportion of 20.6% (16.6–25.2) in Period 1, and adjusted ORS coverage proportion of 50.5% (43.2–57.9) in Period 2

* = *p* < 0.05, ** = *p* < 0.01, *** = *p* < 0.001, **** = *p* < 0.0001

**Table 3:** Change in national and subnational prevalence of ORS use for Demographic Health Survey Cycles: Zambia 2007 vs. 2013/14

Zambia	2007, *N* = 746	2013/14, *N* = 1928	Change	^a^*p*-value
	P1% (CI)	P2% (CI)	P1 - P2	
**Overall**	60.8 (56.1–65.3)	64.7 (61.8–67.5)	3.7	0.156
**Province**				
Central	56.4 (40.1–71.4)	58.9 (49.3–67.9)	2.5	0.795
Copperbelt	63.4 (50.1–74.9)	62.1 (55.5–68.3)	−1.3	0.855
Eastern	75.8 (63.9–84.8)	66.3 (57.9–73.8)	−9.5	0.176
Luapula	49.1(34.7–63.6)	73.5(65.7–80.1)	24.4	0.003
Lusaka	55.8 (37.9–72.2)	74.5 (66.9–80.9)	18.7	0.038
Muchinga	–	52.0(42.6–61.2)	–	
Northern	56.1(45.9–65.9)	56.1(47.1–64.8)	0.0	0.9998
Northwestern	65.3(52.3–76.3)	65.5(57.2–73.0)	−0.2	0.977
Southern	61.6(51.0–71.2)	65.4(55.0–74.4)	2.8	0.604
Western	59.4(44.7–72.7)	65.7(55.5–74.6)	6.3	0.478
**Location**				
Rural	61.6(56.0–66.8)	62.8(58.9–66.6)	1.2	0.715
Urban	59.3(50.4–67.6)	67.8 (63.6–71.7)	8.5	0.186
**Mother’s age**				
< 25 years	64.7 (58.0–70.8)	67.4 (62.9–71.6)	2.7	0.785
≥ 25 years	58.7(52.8–64.3)	63.1 (59.4–66.6)	4.4	0.695
**Education**				
< high school	61.0(55.7–66.0)	63.4(59.7–66.9)	2.4	0.895
≥ high school	60.5 (51.1–69.1)	67.2 (62.8–71.3)	6.7	0.495
**HIV status**				
HIV positive	55.3 (44.5–65.7)	67.7 (59.5–74.9)	12.4	0.065
**Wealth Index (Quintile)**				
Lowest (1)	62.5(52.6–71.4)	60.9(55.1–66.4)	−1.6	0.781
Second (2)	61.3(52.4–69.5)	65.4(59.5–70.9)	4.1	0.423
Middle (3)	56.5(47.6–65.1)	60.4(54.6–66.0)	−3.9	0.462
Fourth (4)	62.6(54.9–69.8)	69.7(63.3–75.3)	−7.1	0.147
Highest (5)	60.0(43.2–74.8)	68.3(60.5–75.1)	8.3	0.345

^a^Based on a two-sided Chi-squared test

**Table 4:** Change in national and subnational prevalence of ORS use for Demographic Health Survey Cycles: Malawi 2010 vs. 2015/16

Malawi	2010, *N* = 869	2015/16, *N* = 1153	Change	^a^*p*-value
	P1% (CI)	P2% (CI)	P1 - P2	
Overall	72.3 (68.4–75.9)	64.6 (60.9–68.1)	−7.7	0.004
**Province**				
Northern region	78.4 (67.386.5)	61.9 (51.5–71.3)	−16.5	0.023
Central region	73.4 (67.5–78.5)	63.2 (57.1–68.9)	−10.2	0.015
Southern region	69.9 (63.6–75.4)	66.7 (62.0–71.0)	−3.2	0.402
**Location**				
Rural	72.3 (68.4–75.9)	65.6 (61.8–69.1)	−6.7	0.013
Urban	72.0 (55.0–84.4)	58.9 (48.9–69.9)	−13.1	0.015
**Mother’s age**				
< 25 years	68.2 (62.1–73.7)	61.2 (55.9–66.3)	−7.0	0.004
≥ 25 years	75.2 (70.2–79.6)	67.4 (62.0–72.3)	−7.8	0.003
**Education**				
< high school	71.2 (67.1–75.1)	64.2(60.0–68.1)	−7.0	0.005
≥ high school	78.1 (67.9–85.8)	66.1(57.1–74.0)	−12.0	0.012
**HIV status**				
HIV positive	80.5 (68.6–88.6)	60.2 (47.3–71.8)	−20.3	0.019
**Wealth Index (Quintile)**				
Lowest (1)	68.9(61.5–75.5)	59.4(51.5–66.9)	−9.5	0.078
Second (2)	75.5(67.2–82.3)	64.2(56.7–71.0)	−11.3	0.040
Middle (3)	69.0(60.5–76.5)	65.9(57.6–73.3)	−3.1	0.579
Fourth (4)	74.4(66.4–81.1)	69.9(61.8–76.9)	−4.5	0.403
Highest (5)	74.9(62.5–84.2)	66.6(54.0–77.2)	−8.3	0.320

^a^Based on a two-sided Chi-squared test

### Subnational prevalence of ORS use

For Zimbabwe, substantial increases in ORS coverage ranging from 10.3 to 44.2%, were observed in 7 of the 10 provinces. The largest percentage increases were observed in Matabeleland South (44.2%) from 12.3%(95% CI: 5.4–25.7) to 56.5%(39.5–72.1); followed by Bulawayo (39.9%) from10.4(4.4–22.8 to 50.3(33.7–66.8); and Midlands had the lowest increase (10.3%) from 28.4(19.0–40.3) to 38.7(29.1–49.3). Of the 10 provinces in Zambia, Luapula (24.4%) from 49.1(34.7–63.6) to 73.5(65.7–80.1); and Lusaka (18.7%) from 55.8 (37.9–72.2) to 74.5 (66.9–80.9) recorded substantial increases between the two periods. All the three regions of Malawi recorded decreases; the Northern (− 16.5%) from 78.4% (67.386.5) to 61.9% (51.5–71.3); Central, (− 10.2%) from 73.4% (67.5–78.5) to 63.2% (57.1–68.9); and Southern (− 3.2) from 69.9% (63.6–75.4) to 66.7% (62.0–71.0) (Tables 1, 3, and 4).

### Prevalence and change in prevalence of ORS coverage by quintiles of household wealth index

Zimbabwe’s prevalence of ORS in the 2nd Period increased with increasing quintiles of household wealth index, i.e., + 11.9%, + 16.8%, + 26.7%, + 26.4%, and + 16.6%, respectively. For all quintiles of household wealth index there was a statistically significant positive linear increase in the change in ORS coverage (all three trend *p* < 0.0125). In Malawi, there was a statistically significant negative linear decrease in the change in ORS coverage (all three trend p < 0.0125). There was substantial decrease in prevalence across quintiles of household wealth index, i.e., − 9.5%, − 11.3%, − 3.1%, − 4.5%, and − 8.3%, respectively. However, there was no linear trend observed in ORS prevalence change across quintiles of household wealth index in Zambia, i.e., − 1.6%, 4.1%, − 3.9%, − 7.1%, and 8.3%, respectively (Tables 1, 3, and 4).

### Prevalence of ORS use by mother’s HIV status

For Zimbabwe, the mother’s HIV status (+) was associated with ORS use: an increase of 18.2% from 22.8% (14.8–33.4) in 1st Period to 41.0% (31.1–51.8) in 2nd Period. For Malawi, the association between ORS prevalence and the Mother’s HIV status (+) was associated with a 20.3% decrease in ORS use: from 80.5% (68.6–88.6) to 60.2% (47.3–71.8). HIV status was not associated with ORS prevalence in Zambia, 60.5% (51.1–69.1) to 67.2% (62.8–71.3) (Tables 1, 3, and 4).

### Prevalence of ORS coverage by urban vs. rural locations, mother’s age, education, and HIV status

In Zimbabwe, the increases in ORS use stratified by mother’s age, education, and urban vs. rural locations in Period 2 from Period 1 were generally similar to the overall national levels of 21.0% (17.4–24.9) to 40.5% (36.5–44.6). The changes for Zambia were similarly flat and much like the overall national level, 60.8% (56.1–65.3) to 64.7% (61.8–67.5). For Malawi, the decreases in proportions were substantial but also mirrored the overall national decrease, 72.3% (68.4–75.9) to 64.6% (60.9–68.1) (Tables 1, 3, and 4).

Unadjusted and multivariable analyses indicate that the odds varied by sub-regions. For all countries, no notable differences were observed in the aOR for rural vs. urban, mother’s age, education, or HIV status. The aORs for ORS use was 3.95(2.66–5.86) for Zimbabwe, 2.83 (2.35–3.40) for Zambia, and, 0.71(0.59–0.87) for Malawi, suggesting that the odds for ORS coverage for Zimbabwe and Zambia increased significantly in the 2nd period compared to the 1st Period. The corresponding adjusted ORS use proportions in the 1st and 2nd Periods were 20.6% (16.6–25.2) up to 50.5% (43.2–57.9) for Zimbabwe; 42.3%(0.37.7–47.0) up to 67.4%(63.7–71.0) for Zambia; and 72.8% (68.0–77.0) down to 65.7% (60.7–70.3) for Malawi (Tables 2, 5, and 6).

**Table 5:** National and subnational odds ratios for ORS use for Demographic Health Survey Cycles: Zambia 2007 vs. 2013/14

Zambia	2007, N = 746		2013/14, N = 1928	
	Crude OR(CI)	Adjusted OR(CI)	Crude OR(CI)	Adjusted OR(CI)
Overall	Ref	Ref	2.95(2.47–3.52) ^a^	2.83 (2.35–3.40)^a^
**Province**				
Central	0.75(0.31–1.81)	0.80(0.32–1.98)	1.93(1.13–3.31)*	1.98(1.15–3.40)*
Copperbelt	0.56(0.25–1.25)	0.59(0.27–1.30)	1.69(1.07–2.68)*	1.98(1.21–3.23)**
Eastern	0.31(0.13–0.70)**	0.32(0.14–0.74)**	1.41(0.84–2.36)	1.43(0.85–2.41)
Luapula	Ref	Ref	Ref	Ref
Lusaka	0.77(0.30–1.96)	0.83(0.32–2.15)	0.95(0.56–1.60)	1.14(0.66–1.97)
Muchinga	–	–	2.56(1.51–4.35)***	2.57(1.52–4.36)**
Northern	0.75(0.37–1.55)	0.78(0.38–1.61)	2.17(1.29–3.64)**	2.15(1.28–3.60)**
Northwestern	0.51(0.23–1.15)	0.54(0.24–1.19)	1.46(0.88–2.43)	1.54(0.92–2.58)
Southern	0.60(0.29–1.26)	0.64(0.30–1.36)	1.47(0.83–2.60)	1.53(0.87–2.70)
Western	0.66(0.28–1.53)	0.66(0.28–1.55)	1.45(0.82–2.56)	1.48(0.84–2.59)
**Location**				
Rural	0.91-(0.59–1.39)	0.66(0.38–1.16)	1.25(0.97–1.60)	1.05(0.75–1.47)
**Mother’s HIV status**				
HIV positive	1.30(0.81–2.07)	1.30(0.78–2.16)	0.86(0.59–1.26)	0.89(0.60–1.32)
**Wealth Index (Quintile)**				
Lowest (1)	0.90(0.41–2.00)	1.66(0.64–4.28)	1.38(0.92–2.09)	1.19(0.65–2.18)
Second (2)	0.95(0.44–2.06)	1.55(0.62–3.88)	1.14(0.75–1.73)	1.05(0.59–1.87)
Middle (3)	1.16(0.53–2.50)	1.67(0.70–3.96)	0.41(0.93–2.13)	1.30(0.76–2.21)
Fourth (4)	0.90(0.43–1.87)	1.02(0.48–2.15)	0.94(0.58–1.51)	0.95(0.57–1.58)
Highest (5)	Ref.	Ref.	Ref.	Ref.
**Mother’s age**				
< 25 years	0.78(0.55–1.10)	0.86(0.59–1.24)	0.83(0.65–1.06)	0.82(0.63–1.07)
≥ 25 years	Ref	Ref	Ref	Ref
**Education**				
< high school	0.98(0.64–1.50)	0.99(0.64–1.52)	1.18(0.93–1.51)	1.04(0.78–1.40)
≥ high school	Ref	Ref	Ref	Ref

^a^Corresponding to adjusted ORS coverage proportion of 42.3% (0.37.7–47.0) in Period 1, and adjusted ORS coverage proportion of 67.4% (63.7–71.0) in Period 2

* = *p* < 0.05, ** = *p* < 0.01, *** = *p* < 0.001, **** = *p* < 0.0001

**Table 6:** National and subnational odds ratios for ORS use for Demographic Health Survey Cycles: Malawi 2010 vs. 2015/16

Malawi	2010, N = 869		2015/16, N = 1153	
	Crude OR(CI)	Adjusted OR(CI)	Crude OR(CI)	Adjusted OR(CI)
**Overall**	Ref	Ref	0.71(0.59–0.86) ^a^	0.71(0.59–0.87) ^a^
**Province**				
Northern region	0.64(0.34–1.20)	0.61(0.32–1.16)	1.23(0.77–1.97)	1.19(0.73–1.96)
Central region	0.84(0.56–1.25)	0.82(0.54–1.23)	1.16(0.84–1.61)	1.09(0.79–1.51)
Southern region	Ref	Ref	Ref	Ref
**Location**				
Rural	0.98(0.46–2.12)	0.92 (0.47–1.79)	0.78 (0.49–1.26)	0.59(0.35–1.01)
Mother’s HIV status				
HIV positive	0.61 (0.32–1.16)	0.64 (0.33–1.24)	1.25 (0.72–2.16)	1.21(0.69–2.11)
**Wealth Index (Quintile)**				
Lowest (1)	1.34(0.69–2.62)	1.15(0.63–2.12)	1.36(0.72–2.58)	1.71(0.84–3.48)
Second (2)	0.97(0.48–1.93)	0.76(0.39–1.48)	1.11(0.60–2.07)	1.37(0.70–2.69)
Middle (3)	1.34(0.67–2.67)	1.14(0.59–2.18)	1.03(0.55–1.94)	1.25(0.63–2.48)
Fourth (4)	1.02(0.51–2.07)	0.89(0.47–1.72)	0.86(0.44–1.67)	0.95(0.47–1.90)
Highest (5)	Ref.	Ref.	Ref.	Ref.
**Mother’s age**				
< 25 years	1.41(0.99–2.02)	1.48(1.01–2.17)*	1.31(0.94–1.82)	1.29(0.92–1.79)
≥ 25 years	Ref	Ref	Ref	Ref
**Education**				
< high school	1.44(0.83–2.50)	1.49(0.84–2.64)	1.09(0.71–1.67)	1.05(0.64–1.72)
≥ high school	Ref.	Ref.	Ref.	Ref.

^a^Corresponding to adjusted ORS coverage proportion of 72.8% (68.0–77.0) in Period 1, and adjusted ORS coverage proportion of 65.7% (60.7–70.3) in Period 2

* = *p* < 0.05, ** = *p* < 0.01, *** = *p* < 0.001, **** = *p* < 0.0001

We further stratified ORS coverage for each period comparing rural vs. urban locations and according to the mother’s age, high school completion and HIV status. In Zimbabwe, Period 1 results for urban Manicaland show the highest ORS coverage 44.6% (25.3–67.8), more than double the national average. Older mothers had higher proportions of ORS use in 5 of the 10 provinces. There was greater use of ORS by mothers with at least high school education; however, only in Mashonaland West do results show higher ORS use among mothers without high school education. For Zambia, higher ORS coverage was observed in rural locations of the 7 out of the nine provinces excluding Northern and Southern provinces where urban locations had higher propensity. Younger mothers had higher proportions of ORS use in all provinces except Southern and Western provinces where older mothers had higher propensity to use ORS, However, except for Lusaka and Southern provinces, all other provinces had greater propensity for ORS use among HIV positive mothers. In Period 1, the Southern region of Malawi’s three provinces had higher ORS use in rural locations mostly. Older mothers, mothers who had completed high school, and HIV positive mothers had higher ORS use in all the three regions comparatively. (Supplemental Table 1).

Stratified analysis by urban vs. rural, mother’s characteristics revealed cascading country-specific patterns that were not uniform across the three countries under study. Common in both periods was the fact that lack of high school education among mothers was associated with lower coverage. In Period 1, being in a rural location and the mother’s lack of high education in general had lower ORS coverage than urban locations for all three countries. During the same period, younger mothers and HIV- mothers had lower ORS use in Zimbabwe and Malawi, it was older mothers and HIV positive mothers in Zambia who had lower ORS coverage. For Period 2, urban ORS coverage exceeded 50% in 5 of the 10 provinces of Zimbabwe. Both urban locations of Harare and Masvingo had coverage of 55.9% (26.6–81.6). Rural locations of Mashonaland East and Harare had the lowest coverage (under 32%). Six of the 10 provinces of Zimbabwe had lower ORS coverage among older mothers compared to younger mothers. In five of the 10 provinces in Zimbabwe, HIV positive mothers had higher ORS coverage compared to HIV- mothers. For Period 2, ORS coverage was lower in rural locations in three of the10 provinces of Zambia compared to urban locations. Younger mothers had lower ORS coverage in six provinces of Zambia, however, they achieved greater than 80% ORS coverage in Lusaka and Luapula provinces. In 7 of 10 subnational regions of Zambia, mothers who lacked high school education, or the mothers with a negative HIV status had lower coverage of ORS compared to mothers with more education, or whose HIV status was positive. In Malawi, there was an acute urban/rural divide for Period 2, where the coverage in rural locations for all the three provinces was 6- to 12-fold lower than urban locations. With respect to mother’s age, education, and HIV status, results indicate that the mothers’ lack of high school education, younger mothers and the HIV positive mothers had lower ORS coverage compared to older mothers, mothers with high school education, and those whose HIV status was negative. In general, Period 2 results show a more diverse mix for each country. In Zimbabwe, rural locations, older mothers, lack of high school education and HIV negative mothers had lower ORS coverage. For Zambia, on the other hand, rural locations, older mothers, lack of high school education and HIV positive mothers had lower coverage comparatively. In Malawi, rural locations, younger mothers, lack of high school education and HIV positive mothers was more inclined to lower ORS use compared to mothers being older, had high school education and were HIV- (Supplemental Table 2).

## Discussion

This paper had a specific focus: to examine change in patterns of ORS coverage over two DHS Cycles in three countries of Zimbabwe, Zambia and Malawi to help identify subnational areas in need of targeted interventions. Study results present mixed experiences at national, subnational and covariate levels. At national levels, ORS use doubled in Zimbabwe, suggesting considerable progress. ORS coverage decreased in Malawi but did not change in in Zambia. At the subnational levels, ORS use increased in Matebeleland North and Bulawayo provinces of Zimbabwe. In Zambia, ORS use increased in Lusaka and Luapula provinces, whereas in Malawi ORS use decreased in all the provinces. These findings reinforce that national estimates mask the coverage of ORS at local level, the level at which health policies need to be implemented. Such local-level estimates of ORS coverage are useful in identifying vulnerable sub-populations most in need of increased efforts to prevent child mortality.

Our study is one of the first to explore subnational coverage of ORS and the first attempt to examine patterns of the change in ORS use over two DHS Cycles to help identify areas in need of targeted interventions. Data summaries at local levels provide an opportunity to further explore the granularity of estimates and to reveal nuances hidden by aggregated national data. This higher resolution will aid governments focus efforts in regions with low coverage. We compare our results to those from previous studies and also discuss the possible explanations for our results.

### Previous studies

Our findings are identical to ORS coverage observed in other countries in SSA. The ORS coverage observed in Zambia and Malawi in 1st Period, is approximately equal to the coverage of 61.1% observed in Namibia in 2000. In the 1st Period, Zambia and Malawi had 2 to 3-fold higher ORS coverage than most SSA countries with coverages below 20% in Madagascar (12.4), Ethiopia (13.1), Rwanda (13.6) Mali (14.0), Chad (15.1), Cameron (16.8), Togo (17.1), Niger (17.6), Nigeria (18.2), and Burkina Faso (18.9). Zimbabwe, with ORS coverage of 21.0% in the 1st Period had ORS coverage roughly equal to Senegal (20.4), Mauritania (22.6), Côte d’Ivoire (23.6), Gabon (24.8), Kenya (29.2). However, the ORS coverage for Zimbabwe of 21.0 in the 1st Period was 1.6 to 3.1 times lower than Uganda, Guinea, Ghana, Eritrea, Mozambique, South Africa, Tanzania, Namibia with coverage ranging from 33.5–61.1% [20]. A 2018 study of Ethiopian mothers reported a prevalence of ORS of 51%, which was lower than the coverage in Zambia and Malawi but higher than the coverage in Zimbabwe [21]. .In 12 SSA countries not included in our study the median ORS coverage in the 1st Period was lower 38% (35–41) [22]. The ORS coverage observed in Zambia and Malawi in 2nd Period, is roughly equal to the coverage of 61.1% observed in Namibia. In the 1st Period, Zambia and Malawi had 2–3 fold higher coverage than most SSA countries with coverages below 20% in Madagascar (12.4), Ethiopia (13.1), Rwanda (13.6) Mali (14.0), Chad (15.1), Cameron (16.8), Togo (17.1), Niger (17.6), Nigeria (18.2), and Burkina Faso (18.9). Zimbabwe, with ORS coverage of 21.0% in the 1st period had ORS coverage to similar to that observed in Senegal (20.4), Mauritania (22.6), Côte d’Ivoire (23.6), Gabon (24.8), Kenya (29.2). However, the ORS coverage for Zimbabwe of 21.0 in the 1st Period was 1.6 to 3.1 times lower than Uganda, Guinea, Ghana, Eritrea, Mozambique, and South Africa. In the same period some countries, Tanzania, Namibia had higher ORS coverage ranging from 33.5–61.1% [20]. The median ORS coverage in 12 SSA countries with high burdens of childhood diarrhea was 38%(35–41) suggesting that Zimbabwe, Zambia and Malawi had higher ORS coverage in both periods. Only Sierra Leone had the highest ORS coverages at 85% (83–87) [22].

### Morbidity and mortality attributable to diarrhea

The decline in ORS coverage in Malawi and the stagnation in Zambia should be viewed alongside patterns of diarrhea morbidity in SSA during the period overlapping the DHS cycles we studied. A DHS study comparing diarrheal morbidity in SSA countries (Burkina Faso, Mali, Nigeria, and Niger) revealed that the proportion among under-5 children varied considerably across the cohorts of birth from 10 to 35%. Relative to 1990–1994 cohort of children < 5 years, diarrheal morbidity declined by half in the 2000–2004 cohort and by 75% in the 2010–2015 cohort [23].

Modeling estimates from the Institute for Health Evaluation and Monitoring (IHME) reveal that rates of diarrheal morbidity increased from 33.1 per 1000 children < 5 years in 2000 to 41.6 in 2015. In Malawi during the same period the diarrheal prevalence rate also increased from 45.5 to 57.6 [24]. However, in Zambia, the rate decreased from 50.5 to 40.2. (see https://vizhub.healthdata.org/lbd/diarrhoea). It would be natural to expect a country such as Zimbabwe, which experienced an increase in morbidity to record higher levels of ORS use. It would also be intuitive for a country in which diarrheal morbidity declined like Zambia, to record lower ORS use. The pattern observed in Malawi, with an increase in diarrheal morbidity and an unchanged ORS use is counterintuitive and needs further investigation.

Also, IHME data shown in Fig. 2 shows heterogeneous long-term patterns of deaths rates and prevalence per 100,000 children between 1990 and 2017 in the three countries [24]. .Whereas our study revealed that ORS coverage increased by 19.9% overall in Zimbabwe, coverage remained below 50% nationally and in 7 of 10 provinces. In Zambia and Malawi, on the other hand, coverage was uniformly greater than 50% nationally and in all provinces. The increase between 1st and 2nd Period was minor in Zambia but in Malawi the proportions decreased between 1st and second period. The divergent patterns are in part explained by the peak in cases of diarrhea in Zimbabwe, a period of severe economic downturn. While it may appear that Zambia and Malawi had better coverage, death rates and prevalence were lower in Zimbabwe, experienced a sudden uptick in prevalence and a bump in death rate during the period of study. Fig. 2 shows that Zimbabwe had the lowest mortality rate relative to Zambia and Malawi up until 2005 when there was a sudden sharp spike in mortality between 2006 and 2010 overlapping with the two DHS cycles which we studied [24]. It was during this period that Zimbabwe experienced the first hyperinflation of the twenty-first century [25].

### Climate change

SADC countries experience more intense droughts linked to changes in El Niño/La Niña-Southern Oscillation patterns. This climate change pattern, that occurs across the tropical Pacific Ocean approximately every five years impact drinking-water sources and sanitation. Diarrheal disease topped the list of health effects associated with this climate change patterns [26, 27]. The additional pressure of climate change on health systems is likely to influence the success of most countries to attain the health-related SDGs.

### Economic

Malawi, according to a 2016 UNICEF report, had a weak economic performance, fiscal challenges, a humanitarian crisis and high levels of poverty among other macro-challenges which subsequently worsened the situation of children in 2016 [6]. The depressed economy might have contributed to the ORS prevalence use slump where the country may have focused more on poverty reduction and seemingly pay less attention to areas such as health. In Zimbabwe, wealth, especially the lowest quintile had a significant effect on ORS use in the second period comparatively; in Malawi, the second wealth quintile level was associated with the highest decrease in ORS prevalence while in Zambia, wealth had not much bearing on ORS use for all the periods. For the past 20 years Zambia has been involved in encouraging mothers to ensure their children who are 5 years or younger get all vaccines, have access to medical care and have their growth monitored. These encouragements potentially helped to increase ORS coverage.

The period 2006–2010 coincided with hyperinflation in Zimbabwe. During the height of inflation from 2008 to 2009, Zimbabwe’s peak inflation was estimated at 79.6 billion percent month-on-month, 89.7 sextillion percent year-on-year in mid-November 2008 [25]. Only Hungary in 1946 had ever experienced worse stratospheric inflation in the history of the world [25]. The Reserve Bank forced Zimbabwe Stock Exchange to shut down. This stratospheric inflation, caused by political instability, created chaos in all spheres of the economy. Punitive economic stifling measures such as the one passed by the Senate and House of Representatives of the United States of America in Congress referred to as Zimbabwe Democracy and Economic Recovery Act (ZIDERA) of 2001, (https://www.congress.gov/bill/115th-congress/senate-bill/2779/text) reverse any gains made in health and stifle affected countries from attaining SDG goals [28]. While on paper economic sanctions were meant to target politicians, as our study shows, there are deeper and far-reaching health consequences and unnecessary loss of life [29–31].

### Political

The case for Zimbabwe is testimony that political and economic disruptions through economic punitive measures such as economic sanctions reverse gains in health and undermine attainment of SDGs. As a result, the Zimbabwe health sector collapsed, there were massive unemployment, food and fuel shortages which served to fuel the health crises including the spike in death rates due to diarrheal disease in children under 5. As Fig. 2 [15] suggests, it will take longer for Zimbabwe to recover to pre-2005 diarrheal prevalence and death rates, let alone reduce neonatal mortality to at least as low as 12 per 1000 live births and under 5 mortality to at least as low as 25 per 1000 live births. Such measures as ZIDERA do more harm than good and have been shown to harm the poor more than rich individuals [28].

### Strengths and limitations

Our study has several strengths. A multi-stage sampling design based on sampling units from national census allowed us to obtain population-based estimates. The DHS studies have high participation rate which limits the potential for participation bias and non-response bias. The use of standard questionnaires, and standardized data collection and sampling procedures, uniform data structures and coding schemes conducted across countries and cycles is another major strength which provides confidence in comparing coverage proportions within subnational regions and among countries. National-level estimates may obscure substantial heterogeneity at spatial scales such as provincial-level, sub-provincial or district-level, where policy decisions are made and implemented. The subnational focus is also currently one of the best approaches to measuring health.

Despite the strengths, our study is not without limitations. Firstly, the observational design of the surveys limits our findings to associations. Secondly, DHS are based on self-report by the mothers, i.e., there is no mechanism to verify the veracity of the statements made by the mothers. The diarrhea was not verified through clinical records potentially introducing misclassification bias and recall bias since we expect mothers not to forget major childhood illnesses where they needed to seek care [32]. Thirdly, that children had to be alive for the mothers to be interviewed implies that only relatively healthy children were sampled, potentially distorting our findings. Finally, mothers younger than 15 years or older than 49 years were not eligible for the Woman’s Questionnaire because a woman had to be 15–49 years of age. If the propensity to use ORS in the women excluded was different than that of women 15–49 years old, that difference would potentially distort our findings.

### Recommendations

Our results can assist policy makers identify hot spots in need of targeted precision public health efforts to improve ORS coverage and save lives using this simple, cheap, and life-saving therapy, to reduce geographic inequalities in ORS to treat diarrhea, thus reducing mortality in children under 5 years. Subnational surveillance, evaluation, and monitoring should be strengthened to achieve Goal #3.2 of Sustainable Development Goals. Our results should be used as background information to develop integrated strategies that improve diarrheal morbidity and mortality rates on a local level. Subnational regions with low ORS coverage signal lower levels of knowledge of mothers, on whom the efficacy of ORS use in preventing child mortality depend. Reasons for the decline in ORS use, especially when diarrhea rates have not declined or increased, together with new interventions to increase ORS coverage should be investigated. Low levels of ORS coverage likely are indicative of high prevalence of key risk factors. Governments should invest in efforts to improve availability of safe drinking-water and adequate sanitation and hygiene.

### Conclusion

Against a backdrop of decreasing diarrheal disease morbidity, adverse changes in climate, and economic hardships, ORS coverage doubled in Zimbabwe, stagnated in Zambia, but declined in Malawi. Monitoring national and province-level trends of ORS use illuminates geographic inequalities and helps identify priority areas for targeting resource allocation. Provision of safe drinking-water, adequate sanitation and hygiene will help reduce the causes and the incidence of diarrhea. Health policies to strengthen access to appropriate treatments such as vaccines for rotavirus and cholera and promote use of ORS to reduce the burden of diarrhea should be developed and implemented.

## Supplementary Information

**Additional file 1: Supplemental Table 1.** Coverage of ORS by location, and mother’s characteristics, 2007/2010 (1st period). **Supplemental Table 2.** Coverage of ORS by location, and mother’s characteristics, 2013/2016 (2nd Period).

## Data Availability

The data that support the findings of this study are publicly available from https://www.dhsprogram.com/data/available-datasets.cfm.

## Abbreviations

aOR: adjusted Odds Ration
CI: 95% Confidence interval
DALYs: Disability-adjusted life-years
DHS: Demographic health surveys
EA: Enumeration area
EML: Essential medicines list
GAPP: Global action plan for the prevention and control of pneumonia and diarrhea
GBD: Global burden of diseases, injuries and risk factor study
HAART: Highly active antiretroviral treatment
HIV: Human immunodeficiency virus
IRB: Institutional Review Board
LMICs: Low-middle income countries
ORS: Oral rehydration solution
ORT: Oral rehydration therapy
OR: Odds ratios
PPS: Probability proportional to size
SADC: Southern Africa Development Community
SDG: United Nations General Assembly 2030 Agenda for Sustainable Development Goals
SDI: Socio-demographic index
SSA: Sub-Saharan Africa
TFR: Total fertility rate
UN: United Nations
UNICEF: United Nations Children’s Fund
USAID: United States Agency for International Development
UI: Uncertainty interval
WHO: World Health Organization
ZIDERA: Zimbabwe Democracy and Economic Recovery Act
