# Supplementary material for: Oral rehydration solution coverage in under 5 children with diarrhea: a tri-country, subnational, cross-sectional comparative analysis of two demographic health surveys cycles
Source: BMC Public Health. 2020 Nov 16;20:1716. doi: 10.1186/s12889-020-09811-1 (PMC7670726; doi:10.1186/s12889-020-09811-1)
Supplement: Supplementary file 1 — Additional file 1: Supplemental Table 1. Coverage of ORS by location, and mother’s characteristics, 2007/2010 (1st period). Supplemental Table 2. Coverage of ORS by location, and mother’s characteristics, 2013/2016 (2nd Period). [file 12889_2020_9811_MOESM1_ESM.docx]

**Supplemental Table 1:** Coverage of ORS by location, and mother’s characteristics, 2007/2010 (1^st^ period)

| Country |  | Location | | Mother’s age | | Completed High school? | | HIV status | |
| --- | --- | --- | --- | --- | --- | --- | --- | --- | --- |
| Zimbabwe, N=688 | Prevalence% (CI) | Rural | Urban | <25 years | ≥25 years | Yes | No | - | + |
| Overall | 21.0 (17.4-24.9) | 18.4(14.5-23.0) | 25.9(19.2-34.1) | 19.2(14.4-25.1) | 22.2(17.7-27.5) | 14.9(10.0-21.9) | 24.2(19.39-29.0) | 19.9(16.1-24.4) | 22.8(14.8-33.4) |
| Manicaland | 19.5 (11.6-30.7) | 14.7(7.1-27.8) | 44.6(25.3-67.8) | 18.3(9.8-31.7) | 20.5(10.0-37.5) | 22.9(13.6-35.8) | 13.5(3.4-40.3) | 18.2(10.2-30.3) | 29.4(5.6-74.6) |
| Mashonaland Central | 25.1 (15.7-37.7) | 25.9(15.9-39.1) | 11.4(1.4-54.1) | 24.6(11.4-45.4) | 25.5(15.1-39.8) | 14.0(7.7-24.2) | 37.9(19.7-60.3) | 29.4(17.6-44.9) | - |
| Mashonaland East | 22.7 (14.0-34.6) | 22.3(12.7-36.3) | 24.7(15.2-37.4) | 22.7(9.2-45.8) | 22.7(11.4-40.1) | 30.9(18.0-47.5) | 6.7(1.6-24.0) | 27.8(16.4-43.0) | 14.3(2.8-48.7) |
| Mashonaland West | 9.6(4.1-21.2) | 5.3(1.7-15.2) | 22.9(9.2-46.5) | 15.6(6.0-35.1) | 5.9(1.9-17.4) | 14.3(6.9-27.3) | 3.5(0.8-14.8) | 8.7(3.6-19.3) | 12.9(3.5-37.5) |
| Matebeleland North | 21.7 (13.5-32.9) | 22.0(12.2-36.4) | 20.8(9.0-41.0) | 28.1(15.4-45.6) | 17.7(8.8-32.4) | 28.1(16.6-43.3) | 12.6(4.4-31.1) | 17.2(8.2-32.6) | 35.4(12.4-68.0) |
| Matebeleland South | 12.3(5.4-25.7) | 14.3(6.5-28.8) | 4.9(0.3-48.1) | - | 16.2(7.6-31.1) | 14.6(5.0-35.7) | 8.3(1.0-44.3) | 8.1(2.1-26.4) | 30.1(8.2-67.3) |
| Midlands | 28.4(19.0-40.3) | 27.5(16.7-41.9) | 31.7(16.2-52.8) | 26.7(13.2-46.7) | 29.5(17.7-45.0) | 30.8(20.7-43.1) | 23.3(8.3-50.3) | 23.0(13.0-37.2) | 33.9(12.7-64.5) |
| Masvingo | 14.1(7.2-25.8) | 15.0(7.6-27.2) | - | () | 22.4(11.3-39.6) | 18.5(6.8-41.3) | 10.3(3.7-25.6) | 10.5(4.6-22.3) | 42.5(9.7-83.7) |
| Harare | 27.7(16.5-42.7) | - | 27.7(16.4-42.8) | 20.6(9.4-39.5) | 34.4(18.4-55.0) | 31.4(18.9-47.4) | 6.6(0.7-40.9) | 26.1(14.7-41.9) | 22.7(6.3-56.2) |
| Bulawayo | 10.4(4.4-22.8) | - | 10.4(4.3-23.0) | - | 16.5(7.3-33.0) | 11.5(3.9-29.6) | 7.6(1.0-40.8) | 16.3(5.3-40.0) | - |
| **Zambia, N=746** |  |  |  |  |  |  |  |  |  |
| Overall | 60.8 (56.1-65.3) | 61.6(56.0-66.8) | 59.3(50.4-67.6) | 64.7(58.0-70.8) | 58.7(52.8-64.3) | 60.5(51.1-69.1) | 61.0(55.7-66.0) | 61.7(56.6-66.5) | 55.3(44.5-65.7) |
| Central | 56.4 (40.1-71.4) | 57.4(38.1-74.6) | 52.2(26.9-76.4) | 80.3(59.9-91.7) | 46.2(28.6-64.8) | 57.1(36.7-75.4) | 56.1(36.0-74.5) | 56.5(37.2-74.0) | 52.9(25.9-78.3) |
| Copperbelt | 63.4 (50.1-74.9) | 66.7(46.1-82.4) | 62.5(46.5-76.2) | 74.1(54.3-87.4) | 56.0(36.7-73.6) | 64.6(38.7-84.1) | 62.3(51.7-71.9) | 66.4(52.4-78.0) | 53.1(28.4-76.4) |
| Eastern | 75.8 (63.9-84.8) | 76.9(63.9-86.3) | 63.2(39.1-82.1) | 77.2(58.5-89.1) | 74.8(57.9-86.5) | 80.2(48.9-94.5) | 75.5(62.4-85.2) | 76.6(62.9-86.3) | 70.8(38.8-90.3) |
| Luapula | 49.1(34.7-63.6) | 50.0(33.2-66.8) | 44.4(27.0-63.4) | 52.2(26.7-76.7) | 48.3(32.9-64.0) | 15.3(2.7-54.0) | 53.7(37.6-69.0) | 52.2(37.4-66.6) | 32.3(8.8-70.2) |
| Lusaka | 55.8 (37.9-72.2) | 65.7(44.4-82.2) | 52.5(30.2-73.8) | 61.3(39.8-79.1) | 52.6(33.3-71.2) | 53.1(30.8-74.3) | 57.9(35.6-77.4) | 54.1(32.4-74.4) | 59.8(35.1-80.4) |
| Muchinga | - | - | - | - | - | - | - | - | - |
| Northern | 56.1(45.9-65.9) | 54.1(43.0-64.8) | 69.2(53.9-81.3) | 50.0(33.8-66.3) | 58.9(47.5-69.4) | 44.6(20.9-71.0) | 56.9(45.7-67.3) | 56.9(46.4-66.8) | 23.7(1.6-85.7) |
| Northwestern | 65.3(52.3-76.3) | 68.8(52.6-81.4) | 54.3(32.1-74.9) | 70.6(54.7-82.7) | 60.8(45.0-74.7) | 88.3(77.3-94.3) | 55.9(40.0-70.7) | 67.1(54.1-78.0) | 0 |
| Southern | 61.6(51.0-71.2) | 59.4(46.7-70.9) | 68.6(49.4-83.0) | 49.9(33.3-66.5) | 68.0(56.1-77.9) | 66.1(50.7-78.7) | 59.0(45.2-71.5) | 60.4(48.2-71.4) | 69.7(39.9-88.8) |
| Western | 59.4(44.7-72.7) | 60.3(43.7-74.9) | 52.2(31.0-72.6) | 51.9(28.4-74.6) | 63.7(45.4-78.7) | 44.2(22.1-68.8) | 63.4(44.3-79.1) | 61.0(44.8-75.2) | 48.5(13.5-85.1) |
| **Malawi, N=869** |  |  |  |  |  |  |  |  |  |
| Overall | 72.3 (68.4-75.9) | 72.3(68.4-75.9) | 72.0(55.0-84.4) | 68.2(62.1-) | 75.2(70.2-79.6) | 78.1(67.9-85.8) | 71.2 (67.1-75.1) | 71.5(67.4-75.2) | 80.5(68.6-88.6) |
| Central region | 73.4 (67.5-78.5) | 72.0 (66.2-77.2) | 80.7(57.4-92.9) | 69.6(60.9-77.1) | 76.1(67.8-82.9) | 82.2(68.7-90.7) | 71.3(64.9-77.0) | 73.1(67.1-78.4) | 77.6(48.2-92.8) |
| Northern | 78.4 (67.386.5) | 77.4(65.7-85.9) | 93.6(57.4-99.4) | 81.5(65.1-91.3) | 75.9(61.5-86.1) | 76.9(51.2-91.3) | 78.7(65.9-87.6) | 77.7(66.3-86.1) | 85.8(49.2-97.4) |
| Southern region | 69.9 (63.6-75.4) | 71.5(65.2-77.0) | 54.0(30.1-76.2) | 63.2(53.3-72.2) | 74.1(66.9-80.2) | 71.2(49.8-86.0) | 69.7(63.3-75.4) | 68.2(61.6-74.1) | 80.9(65.4-90.5) |

**Supplemental Table 2**: Coverage of ORS by location, and mother’s characteristics, 2013/2016 (2^nd^ Period).

| Country | Prevalence | Location | | Mother’s age | | Completed High school? | | HIV status | |
| --- | --- | --- | --- | --- | --- | --- | --- | --- | --- |
| **Zimbabwe, N=1014** | N=1014 | Rural | Urban | <25 years | ≥25 years | Yes | No | - | + |
| Overall | 40.5 (36.5-44.6) | 37.8(33.1-42.7) | 46.1(39.1-53.9) | 43.3(36.7-50.1) | 38.9(34.0-44.1) | 44.7(39.9-49.5) | 32.6(26.6-39.1) | 42.2(37.9) | 41.0(31.1-51.8) |
| Manicaland | 36.7 (27.5-47.1) | 37.7(27.4-49.2) | 30.3(16.4-48.9) | 35.0(21.5-51.4) | 37.5(24.5-52.6) | 41.1(30.3-52.9) | 29.6(16.8-46.7) | 39.5(29.0-51.2) | 30.6(7.4-70.9) |
| Mashonaland Central | 40.5 (28.6-53.7) | 39.6(26.9-53.9) | 51.7(38.9-64.3) | 41.2(19.9-66.5) | 40.1(29.3-52.0) | 41.2(27.7-56.1) | 39.9(23.2-59.3) | 41.1(28.5-55.0) | 43.9(17.9-73.8) |
| Mashonaland East | 33.8 (24.1-45.1) | 31.1(20.2-44.5) | 47.9(34.6-61.6) | 44.2(27.5-62.4) | 22.9(11.7-40.0) | 36.1(24.2-50.1) | 30.1(14.7-51.8) | 39.2(27.7-52.0) | - |
| Mashonaland West | 36.1(26.4-47.1) | 34.6(22.8-48.7) | 39.8(24.9-56.8) | 47.8(31.9-64.1) | 29.3(19.3-41.9) | 41.2(31.0-52.1) | 28.3(16.0-45.2) | 39.0(28.9-50.2) | 49.2(21.0-77.9) |
| Matebeleland North | 60.3 (45.8-73.2) | 61.1(45.3-74.8) | 50.8(31.3-70.1) | 70.7(50.5-85.1) | 53.1(30.5-74.5) | 47.5(30.4-65.3) | 78.7(56.3-91.3) | 62.2(46.7-75.5) | 66.2(20.0-93.9) |
| Matebeleland South | 56.5(39.5-72.1) | 58.8(39.3-75.9) | 40.7(17.1-69.5) | 64.0(36.8-84.5) | 49.1(31.0-67.5) | 53.6(30.9-75.0) | 63.3(37.4-83.3) | 55.0(33.6-74.8) | 67.1(31.5-90.0) |
| Midlands | 38.7(29.1-49.3) | 38.9(27.1-52.1) | 38.3(24.1-54.7) | 44.7(30.0-60.5) | 33.5(23.5-45.1) | 45.3(31.3-60.0) | 28.0(14.0-48.0) | 39.2(28.9-50.5) | 31.8(8.8-69.2) |
| Masvingo | 35.1(25.4-46.2) | 33.3(23.4-44.9) | 55.9(26.6-81.6) | 24.8(11.0-47.0) | 40.2(27.2-54.6) | 43.4(30.2-57.6) | 21.5(11.2-37.1) | 33.1(21.8-46.7) | 49.1(26.2-72.5) |
| Harare | 49.4(37.2-61.7) | 31.4(21.8-42.9) | 51.1(37.6-64.4) | 46.5(22.3-72.5) | 50.4(36.9-63.7) | 51.0(38.2-63.6) | 37.4(15.2-66.6) | 52.5(39.0-65.6) | 35.7(14.9-63.8) |
| Bulawayo | 50.3(33.7-66.8) | -- | 50.3(33.6-66.9) | 49.0(26.9-71.5) | 51.4(30.3-72.1) | 53.4(34.6-71.3) | 34.8(9.7-72.5) | 53.1(34.1-71.3) | 31.9(5.5-79.0) |
| **Zambia, N=1928** |  |  |  |  |  |  |  |  |  |
| Overall | 64.7 (61.8-67.5) | 62.8(58.9-66.6) | 67.8(63.6-71.7) | 67.4(62.9-71.6) | 63.1(59.4-66.6) | 67.2(62.8-71.3) | 63.4(59.7-66.9) | 64.3(61.2-67.4) | 67.7(59.5-74.9) |
| Central | 58.9 (49.3-67.9) | 60.0(48.9-70.1) | 55.2(35.9-73.0) | 61.2(47.5-73.4) | 57.1(44.7-68.7) | 62.7(50.1-73.8) | 57.1(45.5-68.0) | 58.8(47.7-69.0) | 60.1(30.0-84.1) |
| Copperbelt | 62.1 (55.5-68.3) | 56.6(45.3-67.2) | 63.6(55.7-70.9) | 60.5(45.6-73.7) | 62.8(52.4-72.2) | 64.6(55.5-72.6) | 59.1(48.9=68.6) | 61.0(53.8-67.8) | 67.3(46.9-82.7) |
| Eastern | 66.3 (57.9-73.8) | 65.9(56.8-74.0) | 71.6(58.2-82.0) | 65.5(52.4-76.6) | 67.0(55.1-77.0) | 60.6(45.7-73.7) | 67.6(58.3-75.7) | 67.9(59.6-75.2) | 39.2(15.1-70.1) |
| Luapula | 73.5(65.7-80.1) | 73.5(64.1-81.1) | 73.6(63.8-81.5) | 80.3(65.4-89.8) | 70.8(61.6-78.6) | 74.9(60.7-85.3) | 73.0(64.6-80.1) | 72.9(64.3-80.1) | 80.5(52.3-94.0) |
| Lusaka | 74.5 (66.9-80.9) | 59.9(42.6-75.1) | 75.9(67.8-82.5) | 81.6(68.1-90.2) | 70.6(60.1-79.3) | 75.4(63.3-84.4) | 73.6(60.8-83.4) | 72.9(64.3-80.0) | 84.3(60.1-95.0) |
| Muchinga | 52.0(46.2-61.2) | 52.6(41.0-63.9) | 49.8(38.9-60.7) | 53.6(38.4-68.2) | 51.1(40.2-61.9) | 65.3(50.4-77.7) | 48.3(37.4-59.3) | 52.3(42.6-61.8) | 44.9(14.7-79.5) |
| Northern | 56.1(47.1-64.8) | 55.2(44.6-65.5) | 60.2(49.1-70.4) | 52.3(40.4-63.8) | 58.0(47.6-67.8) | 57.9(40.3-73.8) | 55.7(45.6-65.4) | 56.0(46.7-64.9) | 57.7(31.7-80.0) |
| Northwestern | 65.5(57.2-73.0) | 68.2(57.9-77.0) | 58.3(47.8-68.1) | 68.9(57.2-78.6) | 62.6(50.6-73.2) | 70.7(56.9-81.6) | 61.8(52.9-70.0) | 66.7(58.1-74.2) | 40.0(16.4-69.3) |
| Southern | 65.4(55.0-74.4) | 64.0(51.4-74.9) | 71.3(58.6-81.4) | 72.0(57.9-82.8) | 61.1(49.7-71.3) | 63.4(49.8-75.1) | 66.4(53.1-75.5) | 63.9(52.0-74.3) | 76.4(60.2-87.4) |
| Western | 65.7(55.5-74.6) | 65.7(53.5-76.1) | 65.3(55.0-74.4) | 73.2(59.7-83.4) | 61.6(48.0-73.7) | 71.2(54.7-83.4) | 64.2(52.5-74.5) | 67.7(56.5-77.2) | 56.8(30.3-79.9) |
| **Malawi, N=1153** |  |  |  |  |  |  |  |  |  |
| Overall | 64.6 (60.9-68.1) | 65.6(61.8-69.1) | 59.9(48.9-69.9) | 61.2(55.9-66.3) | 67.4(62.0-72.3) | 66.1 (57.1-74.0) | 64.2 (60.0-68.1) | 65.3(61.4-69.0) | 60.2(47.3-71.8) |
| Central region | 63.2 (57.1-68.9) | 64.8(58.5-70.6) | 56.9(40.2-72.2) | 58.9(49.8-67.4) | 66.3(52.7-74.3) | 64.2(51.2-75.4) | 62.9(55.7-69.5) | 64.1(57.5-70.1) | 62.1(37.8-81.6) |
| Northern | 61.9 (51.5-71.3) | 66.8(54.9-76.9) | 44.3(28.6-61.3) | 60.4(46.2-73.0) | 63.8(50.0-75.6) | 67.1(50.3-80.4) | 59.9(47.0-71.5) | 62.6(52.1-72.1) | 61.5(19.9-91.2) |
| Southern region | 66.7 (62.0-71.0) | 66.2(61.3-70.8) | 69.6(55.2-81.0) | 63.7(56.7-70.2) | 69.3(62.9-75.0) | 69.3(54.3-81.1) | 66.2(60.9-71.1) | 67.3(62.5-71.8) | 58.4(41.7-73.4) |
